# Delusive Nature of Flip-Flop Sign Secondary to Histoplasmosis

**DOI:** 10.7759/cureus.70334

**Published:** 2024-09-27

**Authors:** Omer Riyadh, Omron Hassan, Yashwanth Yerramalla

**Affiliations:** 1 Internal Medicine, Kansas City University, Joplin, USA; 2 Pulmonology and Critical Care, Freeman Hospital West, Joplin, USA

**Keywords:** flip-flop fungus sign, histoplasmosis, invasive fungal infections, lymphadenopathy, mass lesion lung, pulmonary disease

## Abstract

Histoplasmosis, caused by *Histoplasma capsulatum*, often mimics the appearance of lung cancer on fludeoxyglucose (FDG) positron emission tomography (PET) scans. The “flip-flop fungus,” due to its similar presentation on imaging modalities, can lead to false-positive results and unnecessary invasive testing. We present a 46-year-old male patient with a lung nodule and bilateral hilar lymphadenopathy on an FDG-PET scan who initially presented with chest discomfort. Histoplasma infection was confirmed through Grocott's methenamine silver (GMS) stains on bronchoscopy-guided biopsy specimens. An analysis of additional cases from the literature highlighted the diverse clinical presentations and outcomes associated with this condition that may be mistaken for other pathology on PET scans, which in this scenario is known as the “flip-flop fungus” sign. Despite the rarity of this condition, the accurate interpretation of PET findings greatly improves patient management strategies and avoids unnecessary invasive testing.

## Introduction

In recent years, there has been a significant increase in histoplasmosis infections throughout the United States, particularly concentrated in the southern Midwest states [1]. Histoplasmosis is primarily caused by the dimorphic soil-dwelling fungus *Histoplasma capsulatum*. Upon inhalation, the organism enters the host and undergoes transformation into yeast forms as it travels along the respiratory system, eventually settling in the alveoli [2]. Although most histoplasmosis infections are asymptomatic and resolve without treatment, they can enter a state of inactivity within granulomas, which can mimic the radiological appearance of lung cancer on fludeoxyglucose (FDG) positron emission tomography (PET) [1].

Pulmonary nodules discovered through computed tomography (CT) scans are subsequently assessed further with FDG PET for risk stratification and evaluation for possible malignancy. Due to the similarities between the presentation of lung cancer and *Histoplasma* infection on FDG PET scans, misinterpretation of the PET avidity may lead to unnecessary testing such as biopsies or inadequate treatments. Malignant lung nodules typically exhibit higher FDG avidity compared to granulomatous fungal infections, whereas in *Histoplasma* infections, the draining lymph nodes often demonstrate greater FDG activity than primary nodules [3]. These atypical presentations, known as the flip-flop fungus (FFF) sign, are frequently misinterpreted on FDG PET scans, leading to false-positive results. Such misinterpretations can subject patients to further invasive testing, such as transbronchial or CT-guided biopsy, increasing their potential risks [3].

Suspicious nodules and masses often prompt a comprehensive imaging evaluation, and PET can be particularly useful in distinguishing between malignant and benign lesions. Delays in diagnosis can have significant consequences, emphasizing the importance of accurate interpretation of PET findings in clinical practice [4]. We present a case of the flip-flop sign with a review of the current literature to highlight clinical and radiographic features encountered during evaluation.

## Case presentation

**Figure 1:**
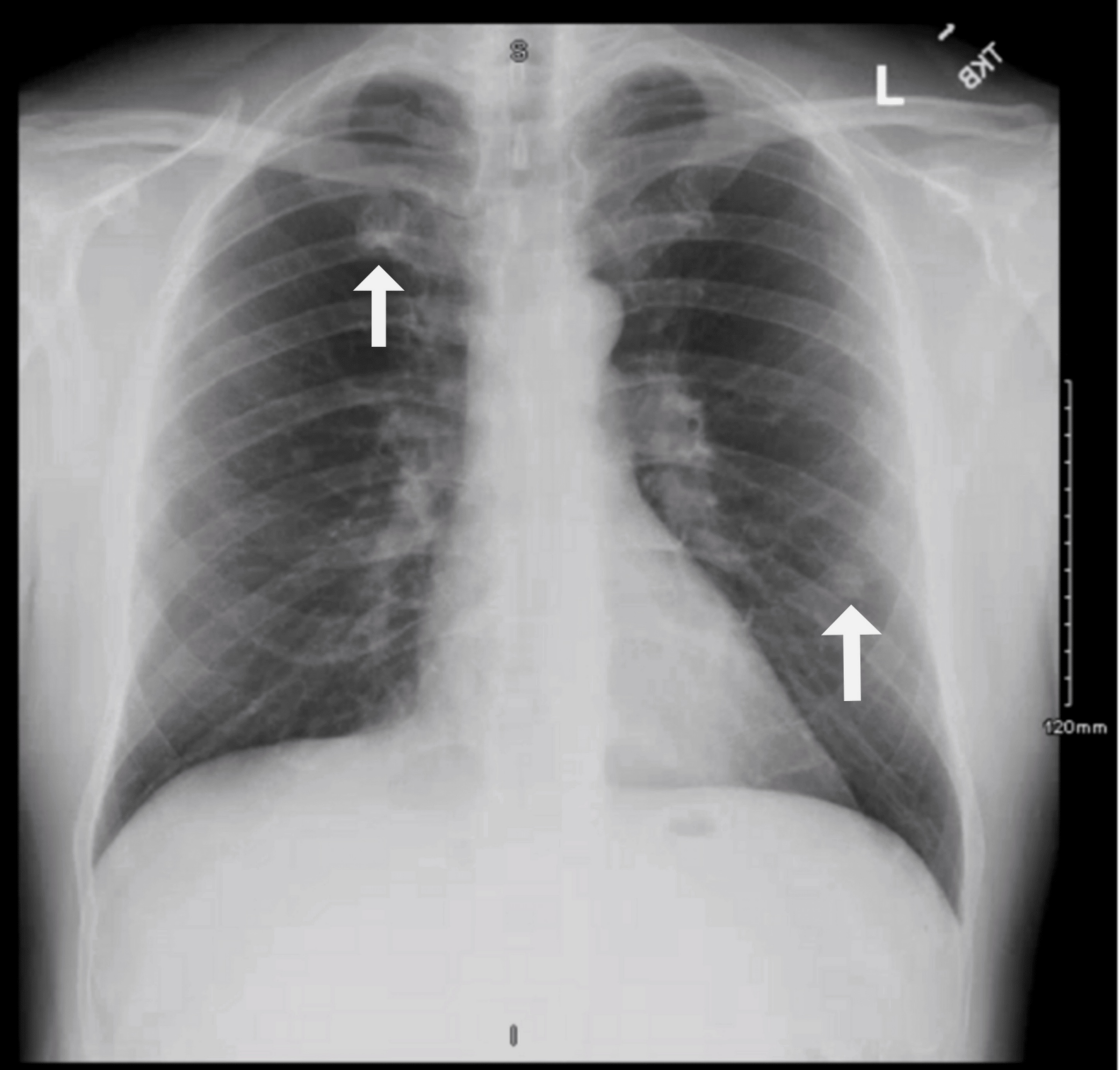
Chest X-ray demonstrating left-sided infrahilar region and right upper lobe nodules

**Figure 2:**
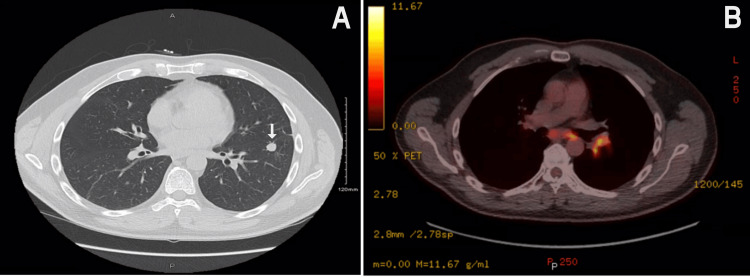
Axial CT scan of the chest without contrast demonstrating left lung mass 11.8 × 11.8 mm (A) and axial PET image demonstrating increased uptake in hilar lymph nodes (B)

A 46-year-old male patient with a past medical history of type 2 diabetes presented to his primary care provider complaining of chest discomfort caused by a lump-like structure over his right lower chest. The patient has a 10-cigarette smoking history in college but has stopped ever since. He also claims to have possibly been exposed to asbestos. The patient did not report any respiratory symptoms. Upon examination, the prominence of his ribs was noted (and no lump was seen), his oropharynx appeared normal, his lungs sounded clear, and there was no evidence of cervical lymphadenopathy. A chest X-ray was performed, revealing a lung nodule in the left lower lobe (Figure 1). Subsequent chest CT confirmed the presence of an 11.8 × 11.8 mm uniformly dense left infrahilar nodule (Figure 2) and an 8.7 × 8.7 mm right upper lobe nodule. Due to suspicion of potential malignancy, a PET scan was conducted, revealing intensely FDG avid mediastinal and bilateral hilar lymphadenopathy with a mass-like opacity in the left infrahilar region along with mildly FDG avid right upper lobe nodule and non-FDG avid right lower lobe nodule. Based on these findings, an endobronchial ultrasound (EBUS) with fine needle aspiration (FNA) of the lymph nodes was performed, suspecting sarcoidosis.

The cytology report indicated the presence of small fragments of lymphoid material amid ciliated bronchial epithelium, along with necrotic cellular material. Grocott's methenamine silver (GMS) stain revealed focal round to oval structures within an area of necrotic hemorrhagic material, suggestive of *Histoplasma* infection. The GMS stain also identified small yeast-like organisms exhibiting narrow-neck budding, while no such organisms were visible on the periodic acid-Schiff stain.

The patient was started on itraconazole after the procedure. A follow-up appointment was scheduled approximately 1.5 months later, during which it was noted that the patient had no cough or symptoms. The dose of itraconazole was subsequently increased due to low blood levels observed (0.44/0.75). In subsequent assessments, antigen testing for *Histoplasma* and *Blastomyces* yielded negative results. Further follow-up was planned, including repeat chest X-rays and surveillance. A repeat chest X-ray conducted three months later showed no abnormalities.

## Discussion

A review of the English-language literature revealed three cases published between 2018 and 2021, which reported initial presentations consistent with the FFF sign. The search revealed a total of three patients who presented with a wide array of symptoms, with chest discomfort being the most common. The majority of histoplasmosis infections are asymptomatic or result in mild flu-like symptoms, which often resolve spontaneously. However, in immunocompromised individuals or in cases of heavy exposure, the infection can lead to severe pulmonary or disseminated disease [2].

Histoplasmosis can present with diverse clinical manifestations, ranging from asymptomatic to severe pulmonary or disseminated disease (Table 1). The radiological appearance of histoplasmosis on FDG PET scans can mimic lung cancer, leading to misinterpretations and false-positive results. Johnson et al. proposed a novel diagnostic paradigm termed the FFF sign, which is based on distinct FDG activity patterns in conjunction with patient history and imaging attributes [3,6]. Particularly evident in cases of benign granulomatous disorders, the FFF sign manifests as a transition of FDG activity from the nodule to the draining lymph nodes during the subacute phase of infection. This phenomenon arises from the ability of the nodule's FDG activity to diminish more rapidly than that of the neighboring lymph nodes, leading to a shift in the epicenter of the highest FDG avidity [6].

**Table 1:** Current case reports and series highlighting flip-flop fungus sign FDG, fludeoxyglucose; PET, positron emission tomography

Publication	Age	Sex	Presenting symptom	FDG PET findings	Initial diagnosis	Outcome
Ruegg et al. (2021) [5]	56	F/1	Chest pain and shortness of breath with no cough	Multiple hypermetabolic lymph node 33 mm in diameter	Autoimmune granulomatous disease	No antifungal treatments were initiated, and the patient's symptoms improved over the next month.
Kraskovsky et al. (2020) [3]	71	M/1	Non-productive cough	Left perihilar nodule measuring 1.6 × 1.1 cm and a 2.6 × 1.7cm prevascular lymph node	-	No treatment was indicated as the patient was asymptomatic and not immunocompromised.
Varghese et al. (2018) [4]	62	F/1	Fevers, chills, drenching night sweats, and profound fatigue	Dense left lower lobe consolidation	Acute infection	-

Malignant tumors typically exhibit higher FDG uptake, while fungal infections tend to have lower FDG avidity [3]. The basis of these differences offers an avenue to distinguish between malignancies and histoplasmosis. Other methods include fungal serology testing, which can significantly influence clinical decision-making and potentially prevent the necessity for invasive interventions.

## Conclusions

The misinterpretation of histoplasmosis as lung cancer on FDG PET scans can have significant clinical implications. False-positive imaging results may lead to unnecessary invasive procedures and subject patients to possible complications and unnecessary expenses. Additionally, delays in accurate diagnosis and appropriate treatment can occur, potentially compromising patient outcomes. Therefore, it is crucial for clinicians to be aware of the FFF sign and consider alternative diagnoses, including histoplasmosis, when interpreting FDG PET scans.
